# Diverse Roles of Akt in T cells

**DOI:** 10.20900/immunometab20210007

**Published:** 2021-01-28

**Authors:** Leena Abdullah, L. Benjamin Hills, Evan B. Winter, Yina H. Huang

**Affiliations:** 1 Department of Microbiology and Immunology, Geisel School of Medicine at Dartmouth, Lebanon, NH 03756, USA; 2 Department of Pathology and Laboratory Medicine, Geisel School of Medicine at Dartmouth, Lebanon, NH 03756, USA

**Keywords:** Akt, T cell differentiation, mTOR, Foxo, thymocytes, CD8 T cells, Th1, Th17, Tfh, Treg

## Abstract

Akt kinases translate various external cues into intracellular signals that control cell survival, proliferation, metabolism and differentiation. This review discusses the requirement for Akt and its targets in determining the fate and function of T cells. We discuss the importance of Akt at various stages of T cell development including β-selection during which Akt fulfills the energy requirements of highly proliferative DN3 cells. Akt also plays an integral role in CD8 T cell biology where its regulation of Foxo transcription factors and mTORC1 metabolic activity controls effector versus memory CD8 T cell differentiation. Finally, Akt promotes the differentiation of naïve CD4 T cells into Th1, Th17 and Tfh cells but inhibits the development of Treg cells. We also highlight how modulating Akt in T cells is a promising avenue for enhancing cell-based cancer immunotherapy.

## INTRODUCTION

Akt kinases, also known as PKB kinases are members of the AGC superfamily of serine/threonine protein kinases and have three isoforms (Akt1, Akt2 and Akt3). All isoforms have high structural similarity, possessing an N-terminal pleckstrin homology (PH) domain, a kinase domain and a C-terminal hydrophobic motif (HM) [1]. Akt1 is ubiquitously expressed [2] while Akt2 is highly expressed in insulin responsive organs such as the liver and skeletal muscles [3], and Akt3 is mainly expressed in the testis and the brain [4]. Although Akt isoforms are largely functionally redundant, distinct isoform-specific functions can be observed in certain circumstances (reviewed in [5]).

**Figure 1.**
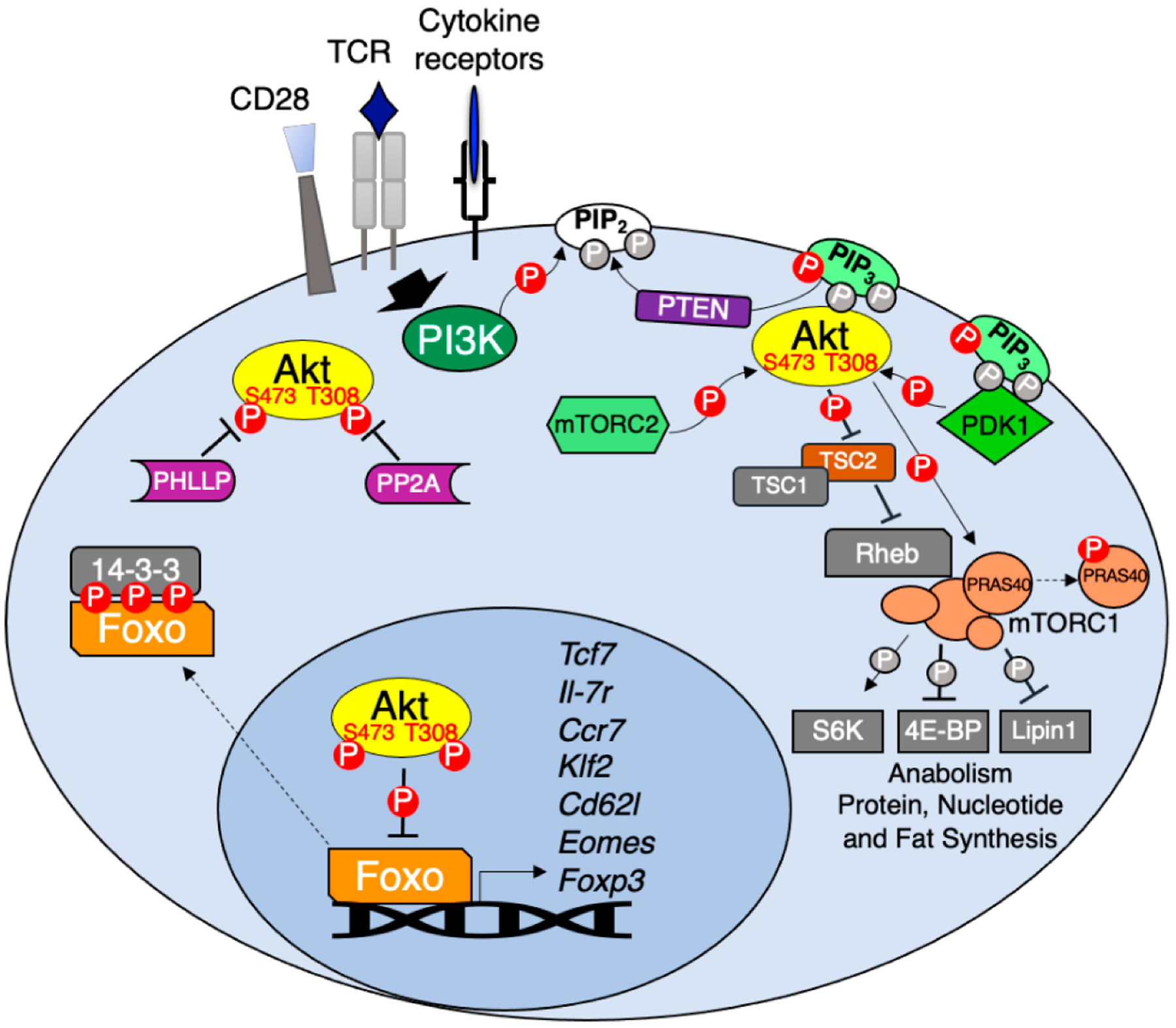
Akt regulation and downstream signaling. TCR, CD28 co-stimulation and various cytokines activate PI3K which in turn phosphorylates membrane PIP_2_ to generate PIP_3_. PIP_3_ acts as the docking site for both Akt and its upstream kinase PDK1 leading to Akt T308 phosphorylation. Full Akt activation occurs following mTORC2 phosphorylation of Akt at S473. Active Akt phosphorylates multiple substrates including the mTORC1 inhibitor TSC2 and PRAS40, resulting in mTORC1-dependent activation of anabolic metabolism. Akt phosphorylation of Foxo transcription factors promotes their association with 14-3-3 adapters leading to Foxo cytoplasmic retention. In the absence of Akt signaling Foxo regulates the expression of genes important for memory CD8 T cell differentiation (*Tcf7, Il7r, Ccr7, Klf2, Sell* and *Eomes*) and Treg development (*Foxp3*). Negative regulators of Akt include PTEN, which dephosphorylates PIP_3_ back to PIP_2_ and protein phosphatases PP2A and PHLPP1/2, which dephosphorylate Akt at T308 and S473, respectively.

**Figure 2.**
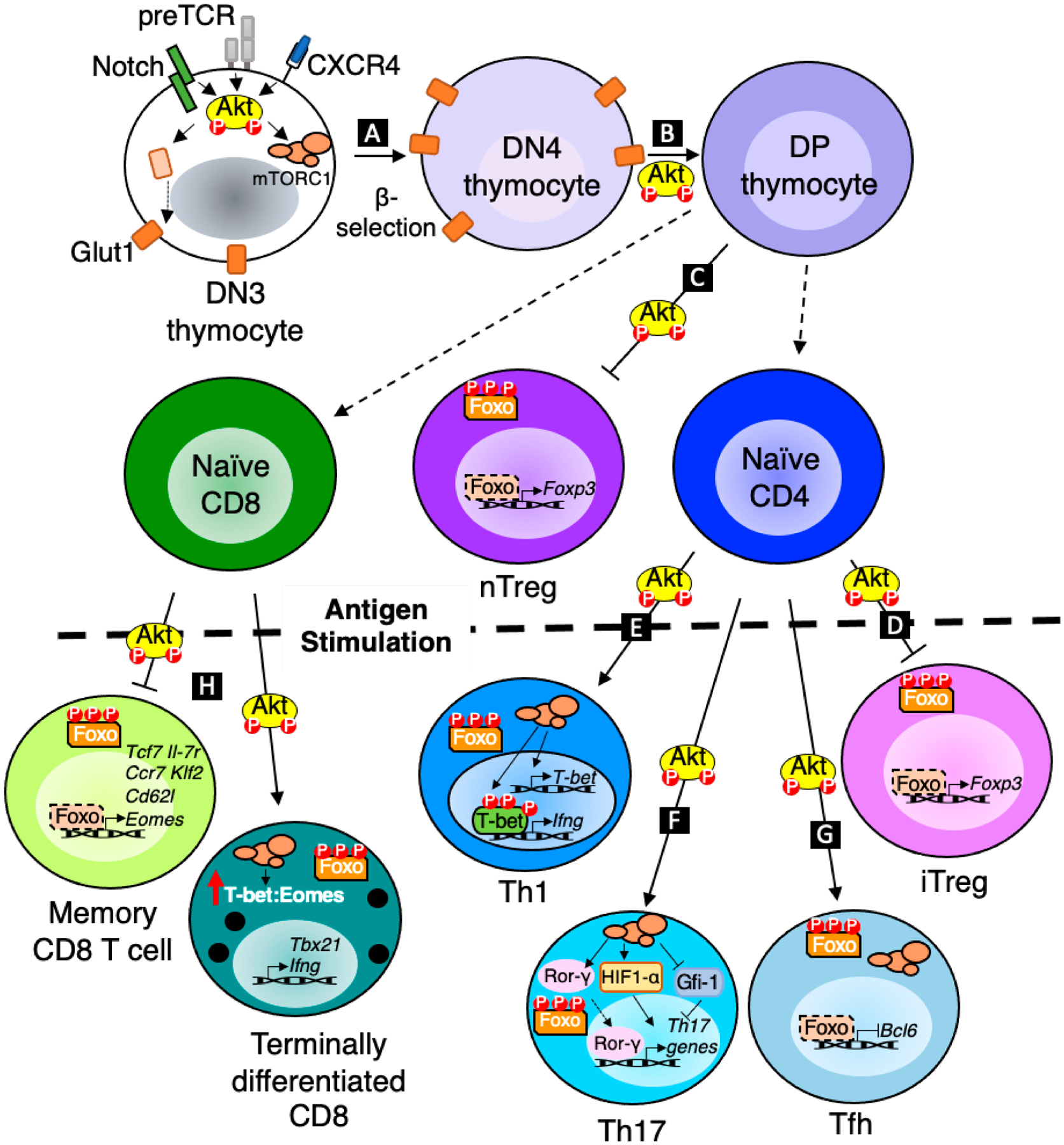
Akt regulation of T cell development, differentiation and function. (**A**) Akt promotes glucose metabolism through upregulation of Glut1 to fulfil energy requirements of proliferating DN3 cells post β-selection. (**B**) Akt also promotes DN4 to DP transition in the thymus. (**C**,**D**) Akt inhibits Foxo1/3 dependent expression of Foxp3 to prevent nTreg and iTreg development. (**E**) Akt dependent mTORC1 activation increases T-bet expression which in turn promotes the transcription of Th1 effector molecules. (**F**) Akt dependent mTORC1 activation positively regulates Rorγ and HIF1α and represses Gfi-1 to promote the transcription of Th17 related genes. (**G**) Akt inhibits Foxo1 to promote Bcl6 expression, and Akt/mTORC1 activity is also required for Tfh development. (**H**) Akt dependent mTORC1 activation and Foxo nuclear exclusion promote effector vs memory CD8 T cell differentiation.

In T cells, TCR and CD28 co-stimulation triggers activation of class I phosphatidylinositol 3-kinases (PI3Ks) leading to the phosphorylation of the 3-hydroxyl group of the inositol ring in the plasma membrane lipid phosphatidylinositol 4,5-bisphosphate (PIP_2_) to generate phosphatidylinositol 3,4,5-trisphosphate (PIP_3_) [6]. PIP_3_ acts as a plasma membrane docking site for the PH domains of Akt [7] and its upstream activator PDK1 [8]. Akt and PDK1 co-localization results in PDK1-mediated phosphorylation of Akt at Threonine (T)308 in its activation T loop [9,10]. For maximum activation, Akt1 requires additional phosphorylation at Serine (S)473 in its hydrophobic motif by mammalian/mechanistic target of rapamycin complex (mTORC)2 [11]. Once activated, Akt phosphorylates multiple downstream targets to regulate cell survival, proliferation, metabolism, and the fate and function of T cells (Figures 1 and 2). Known targets of Akt signaling in T cells include the kinases Gsk3-β and mTORC1, and the transcription factors CREB, Foxo and NF-ĸB (reviewed in [12,13]). However, more recently it has been reported that Akt has differential targets and in turn promotes distinct CD4 T cell fates when phosphorylated only at T308 or at both T308 and S473. Akt activity is countered by the phosphoinositide phosphatase PTEN, which removes the 3-phosphate group of PIP_3_ [14], and the protein phosphatases PP2A and PHLPP1/2, which dephosphorylate Akt at T308 and S473, respectively [15,16].

In this review, we highlight how the signals discussed above (Figure 1) position Akt as a critical regulator of T cell development, differentiation, metabolism, and effector function (Figure 2). In addition, we discuss how the multifaceted roles of Akt in T cells make this kinase an attractive target for improving cancer immunotherapy.

## CONTROL OF EARLY T CELL DEVELOPMENT IN THE THYMUS BY AKT

T cells engage several signaling pathways as they progress through developmental stages in the thymus. Thymocytes in the earliest stages are termed double negative (DN) cells because they lack expression of both CD4 and CD8 co-receptors. DN cells are further subdivided by differential expression of CD44 and CD25 receptors. Multipotent early thymic DN1 cells (CD44^+^CD25^−^) commit to the T cell fate following Notch signaling and differentiate into DN2 cells (CD44^+^CD25^+^) [17]. DN2 cells mature into DN3 cells (CD44^−^CD25^+^). DN3 cells that successfully rearrange and express a unique TCR β chain paired with surrogate pre-Tα chain signal through the pre-T cell receptors (preTCR) to progress through the first checkpoint in thymic development referred to as β-selection [18]. Akt activity is crucial at this checkpoint and is required for the survival, proliferation, metabolism, and differentiation of DN3 thymocytes into DN4 cells (CD44^−^CD25^−^) [19,20]. DN4 cells mature into DP cells (CD4^+^CD8^+^) which in turn can mature into SP cells (CD4^+^ or CD8^+^) following positive and negative selection [21].

All three isoforms of Akt are expressed in thymocytes, but Akt1 and Akt2 are more abundant [22,23]. Loss of any one isoform does not drastically affect thymic development indicating that Akt isoforms have redundant roles in the thymus [22,24]. However, Akt1 deficient mice, which are smaller in body size, do have smaller thymii and thymocytes that are more susceptible to apoptosis [24,25]. Double deficiency in Akt1 and Akt2 leads to a developmental arrest at the DN3 stage where normally cells undergo β-selection, resulting in significantly lower proportions of DN4 and DP cells which become highly susceptible to apoptosis due to insufficient glucose uptake [23]. Ectopic expression of Bcl-xL, which is associated with Akt dependent survival [26], however, is not sufficient to rescue thymic cellularity in the absence of Akt1 and Akt2 [19]. These studies indicate that Akt promotes thymocyte cell survival beyond its control of pro- and anti-apoptotic proteins including Bcl-xL and Bim in thymocytes [26,27].

Akt activity in DN3 thymocytes is not only regulated by preTCR but also by CXCR4 [28,29] and Notch [30–32]. In addition to directing thymocyte localization, the chemokine receptor CXCR4 physically associates with preTCR and acts as a co-activator of preTCR signaling [33]. Antagonism of CXCR4 signaling decreases Akt phosphorylation and results in a developmental block at the DN3 stage [28,29,33]. DN3 cell size, survival, Glut1 expression and glycolysis are also decreased when removed from Notch ligands but can be rescued by ectopic expression of constitutively active Akt [31], suggesting that endogenous Notch signaling activates Akt to support DN3 cell proliferation and metabolism post β-selection. However, in contrast to the traditional PI3K/Akt pathway activated by preTCR and CXCR4, Notch activates Akt indirectly by inducing HES1, which acts to transcriptionally repress expression of PTEN [34]. Together, preTCR, CXCR4 and Notch act cooperatively to control PI3K and Akt kinases during early thymocyte development to promote cell survival by fulfilling the energy requirement of proliferating DN3 cells post β-selection through upregulation of Glut1 receptors.

The effect of Akt on post β-selection thymocytes can be difficult to discern due to developmental defects in DN3 cells. Nevertheless, loss of Akt1 and Akt2 not only led to a block at the DN3 stage but also decreased proliferation of DN4 cells [22], indicating that (pre)TCR-induced Akt activation is required even after β-selection. The ability of constitutively active Akt1 to rescue DN3 cell development to the DP stage in Rag2-deficient cells that are incapable of generating functional TCR chains indicates that Akt also has a prominent role in promoting differentiation of early DN to DP thymocytes [22]. The inability of active Akt1 to promote further maturation to the SP stage [22], however, suggests that Akt is either not required or is insufficient for this developmental process. This notion is supported by observations made when PI3K/Akt activation is antagonized by PTEN over-expression beginning at the DP stage [35]. While DP, SP and peripheral T cell numbers were significantly reduced due to a transitional block before the DP stage, PTEN over-expression did not affect DP cell survival [35]. The inability to detect Akt phosphorylation in normal DP cells along with their high expression of PTEN further suggests that Akt activity is dispensable in DP thymocyte survival [35]. Progression from DP to SP thymocytes requires thymocytes to undergo positive selection, a process that evaluates the ability of newly generated TCRs to appropriately interact with self-peptide MHC complexes, and avoid negative selection of potentially auto-reactive TCRs. While little is known about the role of endogenous Akt activity during selection of DP thymocytes, constitutive expression of active Akt1 enhances positive selection of CD4 T cell through increased MAPK and Lck activity [36]. Further analyses to determine whether Akt contributes in part to DP to SP thymocyte maturation would require conditional ablation of Akt isoforms in DP thymocytes since use of PI3K or PTEN genetic models are complicated by the important contributions of Tec kinases, which are also activated by PI3K and antagonized by PTEN activity (reviewed in [37]).

## REGULATION OF EFFECTOR AND MEMORY CD8 T CELL DIFFERENTIATION BY AKT

After successful development in the thymus, naïve CD8 T cells primed by mature antigen presenting cells undergo activation and clonal expansion and give rise to short-lived effector cells (SLECs) and memory precursor effector cells (MPECs) in the periphery [38]. SLECs provide immediate protection against pathogens but are prone to apoptosis following antigen clearance. MPECs serve as the primary progenitors of long-lived memory cells that produce enhanced secondary responses upon antigen re-encounter. The development of SLECs and MPECs is controlled by integrating signals received through the TCR and various co-receptors and cytokine receptors. Many of these receptors differentially activate Akt kinases to influence the fate decisions made by cytotoxic CD8 T lymphocytes (CTLs) [39].

In contrast to early thymocytes in which Akt promotes global proliferation, survival, or glucose uptake, the major role of Akt in peripheral CD8 T cells is promoting terminal differentiation of activated T cells into SLECs at the expense of memory T cells [40]. These effects are largely due to Akt regulation of two key families of transcription factors: T-box transcription factors and forkhead transcription factors. How Akt regulates these transcription factors in CD8 T cells is described in more detail below.

The T-box transcription factors T-bet and Eomesodermin (Eomes) were initially reported to be interchangeable in CD8 T cells [41]. However, genetic replacement of T-bet with Eomes demonstrated that T-bet is uniquely required for SLEC development [42]. Expression of constitutively active Akt in CD8 T cells increases the ratio of T-bet to Eomes and favors CD8 effector function and terminal differentiation [43]. Akt increases T-bet activity in part by inhibiting the TSC complex to allow Rheb-dependent mTORC1 activation [44]. mTOR kinase increases the T-bet to Eomes ratio and in turn promotes the effector over memory cell fate in activated CD8 T cells [45]. mTOR kinase mainly acts through the mTORC1 complex to regulate CD8 T cell differentiation and loss of mTORC1 activity results in higher proportion of memory precursor CD8 T cells [46]. mTORC1 also independently favors CD8 effector activity by activating ribosomal protein S6 kinase (S6K) and inhibiting eukaryotic translation initiation factor 4E–binding protein (4E-BP) to promote anabolic metabolism to generate lipids, proteins and nucleic acids [47]. Upon activation, CD8 T cells undergo asymmetric division including differential inheritance of mTORC1 activity where the daughter cell proximal to the APC has higher mTORC1 activity and is more glycolytic while the distal daughter cell has lower mTORC1 activity, is less glycolytic, and gives rise to memory CD8 T cells [48].

Akt also skews CD8 responses towards effector T cell differentiation and function by inhibiting the forkhead transcription factors Foxo1 and Foxo3. Phosphorylation of Foxo proteins by Akt triggers Foxo association with 14-3-3 adaptor proteins resulting in their cytoplasmic retention (reviewed in [49]). Foxo1 positively regulates genes required for memory CD8 T cell differentiation, survival, lymphatic trafficking and homeostasis such as TCF1, IL-7R, CCR7, KLF2 and CD62L [50–53] and represses genes important for effector cell differentiation and activity such as Tbet [54] and GzmB [53]. Consequently, Akt activity diminishes the expression of target genes important for memory T cell function and increases genes important for effector T cells. Overexpression of IL-7R is sufficient to rescue MPEC differentiation in CD8 T cells expressing constitutively active Akt [43]. In contrast, expression of an Akt-insensitive Foxo3 is sufficient to abrogate increased IFNg expression in antigen stimulated CD8 T cells that have high Akt activity [40]. Together, these observations demonstrate that Akt-dependent inhibition of Foxo prevents memory T cell differentiation, in part, by preventing IL-7R expression while it simultaneously promotes expression of CD8 T cell effector proteins.

Foxo1 also promotes CD8 memory T cell differentiation and persistence by regulating the expression of TCF1 (*Tcf7*) [53,55]. TCF1 expression levels vary among memory T cell subsets with high TCF1 observed in long-lived central memory T cells [56]. Foxo1 deficiency results in reduced expression of TCF1 [52], which is required for the optimal generation of central memory T cells [57]. In contrast, effector memory T cells, which traffic through peripheral tissues and quickly differentiate into effector cells upon re-activation, express low to intermediate levels of TCF1 [56]. Enforced Foxo1 expression in CD8 memory cells stabilizes TCF1 expression by preventing PRC2-dependent H2K27me3 silencing marks at the *Tcf7* locus [58]. Memory T cell reactivation and expansion during recall responses is also Foxo1-dependent [52,53,55], indicating that Foxo1 activity not only directs the differentiation of memory CD8 T cells, but its continued activity maintains memory T cell identity, longevity and re-activation potential [59–61]. Thus, Akt-inhibition of Foxo1 activity has the potential to impact CD8 memory T cell formation and function at multiple stages of the T cell response. Accordingly, complete loss of Akt activity due to Akt1 and Akt2 deficiency increases central memory T cell differentiation as well as the proliferative capacity of CD8 T cells even following repeat stimulations [62]. However, disrupting PI3K-dependent Akt phosphorylation at Thr308 through expression of a mutant PDK1 hinders the survival of effector T cells as they transition from effector to effector memory T cells [63], indicating that modest levels of Akt activity are required for effector memory T cell differentiation. In contrast, constitutive Akt activity drastically lowers the proportion of MPECs and memory cells, but subsequent pharmacological inhibition of Akt can selectively rescue effector memory cells in vivo [43]. Collectively, these studies reveal the importance of Akt in regulating multiple distinct phases of CD8 effector and memory responses through the control of Tbet, Eomes and Foxo transcription factors whose gene targets promote cell survival, expression of cytokines and cytolytic enzymes and effector or memory T cell differentiation.

## REGULATION OF DIFFERENTIATION OF TH1, TH2, TH17 AND TFH CELLS BY AKT

CD4 T helper 1 (Th1), Th2 and Th17 cells regulate defense against intracellular pathogens, parasites and extracellular pathogens, respectively [64] while T follicular helper cells (Tfh) are specialized in helping B cells undergo immunoglobulin affinity maturation, class switch recombination and differentiation into memory B cells within germinal centers (GC) [65]. The differentiation of naïve CD4 T cells into these T helper subsets is controlled by environmental cues. Specific cytokines trigger distinct signaling pathways to activate lineage-specific transcription factors including Tbet, Gata3, RORγt and Bcl6 to promote Th1, Th2, Th17 and Tfh differentiation, respectively, and is influenced by TCR-induced PI3K and Akt pathways [66–68]. Akt activity promotes Th1, Th17 and Tfh lineages through indirect regulation of Tbet, RORγt and Bcl6 expression but has limited effects on Th2 differentiation.

The ability of Akt to influence CD4 differentiation was first reported in Akt overexpression studies, which showed that Akt promoted IFNg expression in Th1 cells but did not increase Th2 cell specific genes [69]. Akt promotes expression of T-bet via mTORC1 [70]. mTORC1 activity leads to phosphorylation of T-bet at 4 residues that, when partially disrupted, decreases T-bet dependent permissive epigenetic regulation of the IFNg locus and lowers IFNg production [71]. While mTORC1 is a downstream effector of Akt, mTORC2 lies upstream and is responsible for phosphorylating Akt at Serine 473 for full catalytic activity [11]. Genetic ablation of Rictor disrupts mTORC2 and Akt activation, resulting in a defect in both Th1 and Th2 cell differentiation [72]. However, expression of constitutively active Akt only rescues Th1 differentiation [72] suggesting that Rictor/mTORC2-dependent Akt activation is critical for Th1 differentiation. Direct comparison of models that disrupt Rictor (mTORC2) or Rheb (mTORC1) demonstrated that mTORC1 is proximally required for inducing Tbet and RORγt for Th1 and Th17 cell differentiation, respectively [70]. In contrast, disruption of mTORC2 behaves like an mTOR deficient model and demonstrates the importance of mTORC2 in separately promoting Th2 differentiation and in fully activating Akt for Th1 and Th17 differentiation [70,72,73].

Akt regulates Th17 cell differentiation in multiple ways. Akt-induced mTORC1 activation induces transcription factors important for Th17 differentiation and function, HIF1a and RORγt, and inhibits expression of Gfi1, a transcriptional suppressor of Th17 gene targets [74]. mTORC1 promotes HIF1a expression [75], which in turn induces RORγt expression [76]. mTORC1 dependent S6K1 kinase activity is required to inhibit Gfi1 expression while mTORC1 dependent S6K2 kinase binds to RORγ to facilitate nuclear translocation [77]. Together, HIF1a and RORγ promote transcription of Th17 cell specific genes including IL-17 [76] and various glycolytic proteins to help establish Th17 cell identity [75]. Th17 and T regulatory (Treg) cells share common pathways important for their differentiation; however, key signals that favor one fate inhibit the other. Akt is a proximal signal that favors differentiation of Th17 cells at the expense of Treg cells. Casein Kinase 2 (CK2) is a positive regulator of Akt signaling that is important for Th17 differentiation [78,79]. Treatment with CX4945 a pharmacological CK2 inhibitor decreases Akt phosphorylation at both T308 and S473 and favors Treg over Th17 cell differentiation [80,81]. T cells deficient in the CK2 catalytic subunit CK2α have reduced Akt-dependent Foxo1 phosphorylation and consequently higher expression Foxp3 [82]. Moreover, Foxo1 knockdown rescues IL-17A expression and inhibits Foxp3 expression in CK2α-deficient T cells [82]. Thus, CK2 acts to increase Akt phosphorylation of Foxo1 to sway CD4 differentiation towards Th17 cells and away from Tregs.

Tfh differentiation and activity is regulated by graded Akt activity. In comparison to Th1 cells, Tfh cells exhibit decreased Akt T308 and S473 phosphorylation and downstream mTORC1 activity [83]. Further increasing Akt or mTORC1 activity either through IL-2 stimulation or expression of constitutively active Akt favors Th1 differentiation at the expense of Tfh cells in the LCMV viral infection model [83]. Yet, Raptor- (mTORC1), Rictor- (mTORC2) or mTOR-deficient mice exhibit to varying degrees reduced basal and antigen-induced Tfh cells, GC B cell populations and antibody responses, indicating a Tfh requirement for both Akt-dependent mTORC1 and Akt-activating mTORC2 activity [84]. mTORC1 and mTORC2 participate at distinct stages during Tfh differentiation with mTORC1 promoting CD4 T cell proliferation and glycolysis and mTORC2 acting in part through phosphorylation of Akt at S473 [84,85]. Akt activity promotes Tfh development through inhibition of Foxo1, which negatively regulates Bcl6 expression [86]. Ectopic expression of an Akt-insensitive Foxo1 reduces Tfh cell differentiation [86,87], thereby demonstrating that while too much Akt activity diverts CD4 T cells towards Th1 cells the appropriate Akt activity is required for Bcl6 induction during Tfh cell differentiation.

## INHIBITION OF TREG DEVELOPMENT BY AKT

T regulatory cells are a specialized subset of CD4 T cells that express the transcription factor Foxp3 and function to maintain peripheral self-tolerance [88]. Tregs can be subdivided based on their developmental origin; natural Tregs (nTregs) develop in the thymus while induced Tregs (iTregs), differentiate from naïve CD4 T cells in peripheral lymph nodes [89]. The development and function of both nTregs and iTregs are negatively impacted by PI3K/Akt signaling.

As previously described, Akt inhibits nuclear localization of Foxo and transcription of its targets including Foxp3, a necessary transcription factor for Treg identity and function [90]. Foxo-dependent Foxp3 transcription is required during both thymic nTreg development and TCR and TGFβ-induced iTreg differentiation [91,92]. The importance of Tregs was first appreciated in studies linking Foxp3 mutations with the multi-organ autoimmune inflammatory disease observed in scurfy mice and IPEX patients [93–95]. Mice with Treg-specific Foxo1 deficiency phenocopy scurfy mice, highlighting the pivotal role of Foxo1 in Treg function [96]. Surprisingly, Foxo1-deficient Tregs are increased in proportion compared to conventional WT CD4 T cells and retain their ability to suppress T cell proliferation in vitro [96]. However, Foxo1-deficient Tregs are defective in preventing experimental colitis mediated by the transfer of conventional T cells into Rag-deficient mice [96]. This defect can be attributed to aberrant IFNg expression in Foxo1-deficient Tregs since secondary deletion of IFNg rescues Treg-dependent colitis prevention [96]. In contrast, deficiency in both Foxo1 and Foxo3 results in a profound Treg defect including reduced Treg proportions and numbers and an inability to suppress conventional T cells in vitro [91]. This indicates that Foxo transcription factors have partially redundant functions but are collectively required for Treg development and function.

Ectopic expression of constitutively active Akt reduces Foxp3 expression, nTreg development and iTreg differentiation [97]. In contrast, treatment of activated CD4 T cells with Akt or PI3K inhibitors results in higher proportions and levels of Foxp3 expression [98]. Moreover, treatment with PI3K/mTOR inhibitors induces a Treg-like transcriptional profile marked by upregulation of *Ctla4, Foxp3* and down regulation of *Il2* and *Ifng* [98]. An Akt insensitive variant of Foxo3a promotes Foxp3 expression in stimulated CD4 T cells in the presence of TGF-β and increases the percentage of Foxp3^+^ cells [92,99]. In vivo, PTEN expression in Tregs is responsible for physiologically countering PI3K/Akt to maintain Treg stability and suppressive activity [100,101]. Additional studies identified an important upstream requirement for Roquin proteins in maintaining Treg identity through inhibition of the Akt/Foxo axis by regulating PTEN expression and preventing Foxo degradation by Itch [101]. Together, these studies highlight the importance of Akt-mediated regulation of Foxo proteins in the development and function of nTregs and iTregs.

Akt isoforms may have distinct roles in Treg development and function. Akt1 appears to be the predominant isoform that limits Treg function in the setting of autoimmunity. Genetic ablation of Akt1 improves suppression of T cell-mediated CNS disease in an experimental autoimmune encephalomyelitis (EAE) model of multiple sclerosis [102]. In contrast, Akt2 deficiency results in defective Treg suppressive activity and EAE exacerbation [102], indicating that Akt1 and Akt2 isoforms may act in opposition in this context. Unlike mouse Tregs, human Tregs can gain effector activity such as production of the Th1 cytokine IFNg [103]. The generation of Th1-Treg cells requires Akt1-dependent Foxo regulation [104]. Akt1 deficiency allows Th1-Tregs to regain suppressive capacity; however, Akt3 deficiency has the opposite effect [104]. This suggests that while Akt1 checks Treg suppressive activity in both mouse and human, Treg activity is enhanced by Akt2 in mouse and Akt3 in human. One contradictory study, however, identified Akt2 and not Akt1 as the isoform that limits Foxo-dependent Foxp3 induction during iTreg differentiation [105]. Whether or not Akt isoforms are differentially engaged during nTreg and iTreg development or whether distinct Akt isoforms result in a different magnitude or kinetics of downstream signaling remains unresolved.

However, it has recently been suggested that low levels of Akt activity are important for Treg development. Weak TCR signaling that results in phosphorylation of Akt at T308 and to a much lower extent at S473 can promote commitment to the Treg lineage over conventional CD4 subsets [106–108]. Ex vivo stimulated human Tregs show Akt phosphorylation predominantly at T308 [109]. Increasing Akt activity through expression of constitutively activate Akt in human Treg cells causes them to lose their suppressive capacity and instead produce effector cytokines TNFα and IFNg [109]. Proteomic analyses of Akt substrates using mass spectrometry revealed differential target phosphorylation in response to weak vs strong TCR signaling [108]. Following weak TCR signaling, Akt phosphorylates heterogeneous nuclear ribonucleoproteins hnRNP L and hnRNP A1 [108]. Knockdown of both of these ribonucleoproteins diminishes Treg proportions [108]. Akt also differentially regulates metabolites to control Treg fate [110]. In response to weak TCR signaling, Akt selectively phosphorylates and inhibits Citrate synthase (CS), a component of the TCA cycle to promote higher proportions of Foxp3^+^ Tregs [110]. Further analysis revealed that inhibition of CS allows its substrate acetyl-CoA to be used for H3K27 acetylation at the Foxp3 promoter in CD4 T cells [110]. Thus, Akt activity induced by weak TCR signaling can favor differential targets through select phosphorylation of Akt T308 while additional phosphorylation at S473 in response to strong TCR signaling promotes alternative CD4 T cell fates. Further increasing our understanding of the receptors that control the kinetics and magnitude of Akt activity in naïve T cells and how their engagement results in phosphorylation of distinct subsets of Akt substrates to control T cell differentiation will be important to reveal additional mechanistic insight on the regulation of T cell responses.

## PERSPECTIVE

Altogether, it has become apparent that the requirements for Akt activity in T cells depends on its maturation stage, its lineage and its environmental context (Figure 2). The dominant effects of Akt kinase activity on T cells make Akt an attractive target to manipulate in T cell-based immunotherapies. In particular, the importance of generating antitumor CD8 memory T cells has recently been recognized as an important goal for adoptive cell transfer therapy to provide durable protection against tumor recurrence. The ability of Akt to limit memory CD8 T cell formation suggests that pharmacological treatment of CD8 T cells prior to therapy may increase memory T cell differentiation. Preclinical studies using a melanoma mouse model support this strategy, demonstrating that CTLs treated with an Akt inhibitor provided better tumor control and survival [111]. Similarly, pharmacological inhibition of Akt reprogrammed tumor-infiltrating lymphocytes (TILs) isolated from cancer patients to adopt a memory transcriptional and metabolic profile, which improved their longevity when adoptively transferred into NOD scid gamma (NSG) mice [111]. Moreover, CAR T cells generated in the presence of Akt inhibition provided better tumor control and survival when adoptively transferred into mice [112,113]. Similarly, CD8 T cells treated with a variety of Akt inhibitors had a similar transcriptional profile to stem cell memory T cells, high expansion capacity and higher polyfunctionality upon antigen recall [114]. Preventing Akt regulation of Foxo1 may allow TCF1 expression and intratumoral accumulation of stem-like CD8 T cells that are responsive to PD-1 blockade. Thus, combining Akt inhibition with PD-1/PDL-1 blockade may further enhance antitumor responses [115–117]. Several Akt inhibitors are already in clinical trials to inhibit cancer cell survival and proliferation (reviewed in [118]), but here we propose that Akt inhibition combined with cell-based therapies will equip the immune system for better tumor control. However, the complexity of upstream signals that activate Akt coupled with our limited understanding of Akt’s numerous targets highlight the importance for further investigation into the temporal and spatial control of Akt in different T cell subsets to guide the design of personalized therapies.

## ABBREVIATIONS

CCR7: C-C chemokine receptor 7
CS: Citrate synthase
CD62L: L-Selectin
CK2: Casein Kinase 2
CTLs: Cytotoxic CD8 T lymphocytes
DN: double negative (CD4^−^CD8^−^)
DP: double positive (CD4^+^CD8^+^)
Eomes: Eomesodermin
GC: germinal center
IFNg: Interferon gamma
IL-7R: Interleukin-7 receptor
iTregs: induced Tregs
KLF2: Kruppel-like factor 2
MPECs: memory precursor effector cells
mTORC: mammalian/mechanistic target of rapamycin complex
nTregs: natural Tregs
PDK1: Phosphoinositide-dependent protein kinase-1
PH: pleckstrin homology
PI3K: Class I phosphatidylinositol 3-kinases
PIP_2_: phosphatidylinositol 4,5-bisphosphate
PIP_3_: phosphatidylinositol 3,4,5-trisphosphate
preTCR: pre-T cell receptor
PTEN: Phosphatase and tensin homolog
SLECs: short-lived effector cells
SP: single positive (CD4^+^ or CD8^+^)
TCF1: T cell factor 1
TCR: T cell receptor
Tfh: T follicular helper
Th: T helper
Treg: T regulatory
